# Integrated single-cell RNA-seq analysis reveals the vital cell types and dynamic development signature of atherosclerosis

**DOI:** 10.3389/fphys.2023.1118239

**Published:** 2023-03-30

**Authors:** Xiuli Shao, Xiuyang Hou, Xiaolin Zhang, Ruijia Zhang, Rongli Zhu, He Qi, Jianling Zheng, Xiaoling Guo, Rui Feng

**Affiliations:** ^1^ Department of Pharmaceutical Toxicology, School of Pharmacy, China Medical University, Shenyang, China; ^2^ Department of Medical Biotechnology, Liaoning Vocational College of Medicine, Shenyang, China; ^3^ Center of Scientific Research, The Second Affiliated Hospital and Yuying Children’s Hospital of Wenzhou Medical University, Wenzhou, China

**Keywords:** scRNA-seq, atherosclerosis, fibroblasts, fibrosis, remodeling

## Abstract

**Introduction:** In the development of atherosclerosis, the remodeling of blood vessels is a key process involving plaque formation and rupture. So far, most reports mainly believe that macrophages, smooth muscle cells, and endothelial cells located at the intima and media of artery play the key role in this process. Few studies had focused on whether fibroblasts located at adventitia are involved in regulating disease process.

**Methods and results:** In this study, we conducted in-depth analysis of single-cell RNA-seq data of the total of 18 samples from healthy and atherosclerotic arteries. This study combines several analysis methods including transcription regulator network, cell-cell communication network, pseudotime trajectory, gene set enrichment analysis, and differential expression analysis. We found that SERPINF1 is highly expressed in fibroblasts and is involved in the regulation of various signaling pathways.

**Conclusion:** Our research reveals a potential mechanism of atherosclerosis, SERPINF1 regulates the formation and rupture of plaques through the Jak-STAT signaling pathway, which may provide new insights into the pathological study of disease. Moreover, we suggest that SRGN and IGKC as potential biomarkers for unstable arterial plaques.

## 1 Introduction

Atherosclerosis and its thrombotic complications have caused serious diseases such as myocardial infarction, stroke, aneurysm, and disabled peripheral arterial disease (Libby et al., 2019). As a global killer threatening human health and life, atherosclerosis has been increasing in morbidity and mortality. Atherosclerosis is a chronic disease related to inflammation and fibrosis, and a variety of cells are involved in its formation and development (Libby et al., 2011). In the early stage of the disease, after being stimulated by abnormal causes such as hypertension, pro-inflammatory, and high levels of blood lipids, blood leukocytes adhere to the activated endothelium monolayer and migrate to the intima. Monocytes, that recruit the most numerous of the leukocytes, mature into macrophages to adsorb lipids, and then to form foam cells. Followingly, smooth muscle cells migrate from the media to the intima and begin the large-scale proliferation, causing the remodeling of blood vessels and the formation of fibrous plaques. In the advanced period, a large number of plaques accumulate, and then the plaques rupture to form thrombi, which narrow the arteries and block blood flow, resulting in a series of complications, like myocardial ischemia and infarction.

The reported pathological mechanisms of atherosclerosis include abnormal lipid metabolism (Libby et al., 2019), endothelial cells dysfunction, inflammation mediated mainly by macrophages (Koelwyn et al., 2018), and blood vessel remodeling. The endothelial cells in arteries have the anti-inflammatory and maintain the homeostasis of blood vessel inner wall (Gimbrone and García-Cardeña, 2016). Endothelial cell dysfunction promotes abnormal lipids and pro-inflammatory factors to induce inflammation of blood vessels. Macrophages play an important role in the inflammatory response (Ho et al., 2016). They can absorb plentiful lipids, and then accumulate in the vascular intima to form foam cells in the early stage of atherosclerosis. In the pathological state, smooth muscle cells participate in the synthesis of fibrin to remodel blood vessels and form atherosclerotic plaques with fibrous caps (Wirka et al., 2019). Eventually, a large number of plaques gather, and then rupture to produce thrombotic complications.

Fibrosis is involved in blood vessel remodeling in atherosclerosis. Previous studies had shown that in the initial period of atherosclerosis, fibrosis caused the remodeling of endothelial-damaged artery to lead to the initial plaque forming. However, in the later stage of atherosclerosis, inflammation in the plaque deteriorates and fibrosis begins to decrease, resulting in the formation of a thinner fibrous cap, which promotes the rupture of plaques and causes the thrombi (Higashi et al., 2016). Fibroblasts, as a kind of mesenchymal cells, may be one of the main cell groups participating in blood vessel remodeling during the atherosclerosis. Interestingly, fibroblasts are mainly distributed in the adventitia. The fibroblasts in the plaque may be attributed to the migration of adventitial fibroblasts (Shi et al., 1996; Sartore et al., 2001). Fibroblast-like cells are common in atherosclerotic lesions and have been confirmed to be formed by endothelial to mesenchymal transition (EndMT) (Evrard et al., 2016) and smooth muscle cells (SMCs) to fibromyocytes modulation (Wirka et al., 2019). The process of atherosclerosis involves the participation and interaction of multiple type cells. However, the role of fibroblasts interacted with other cell types in different period of atherosclerosis are still unclear.

**FIGURE 1:**
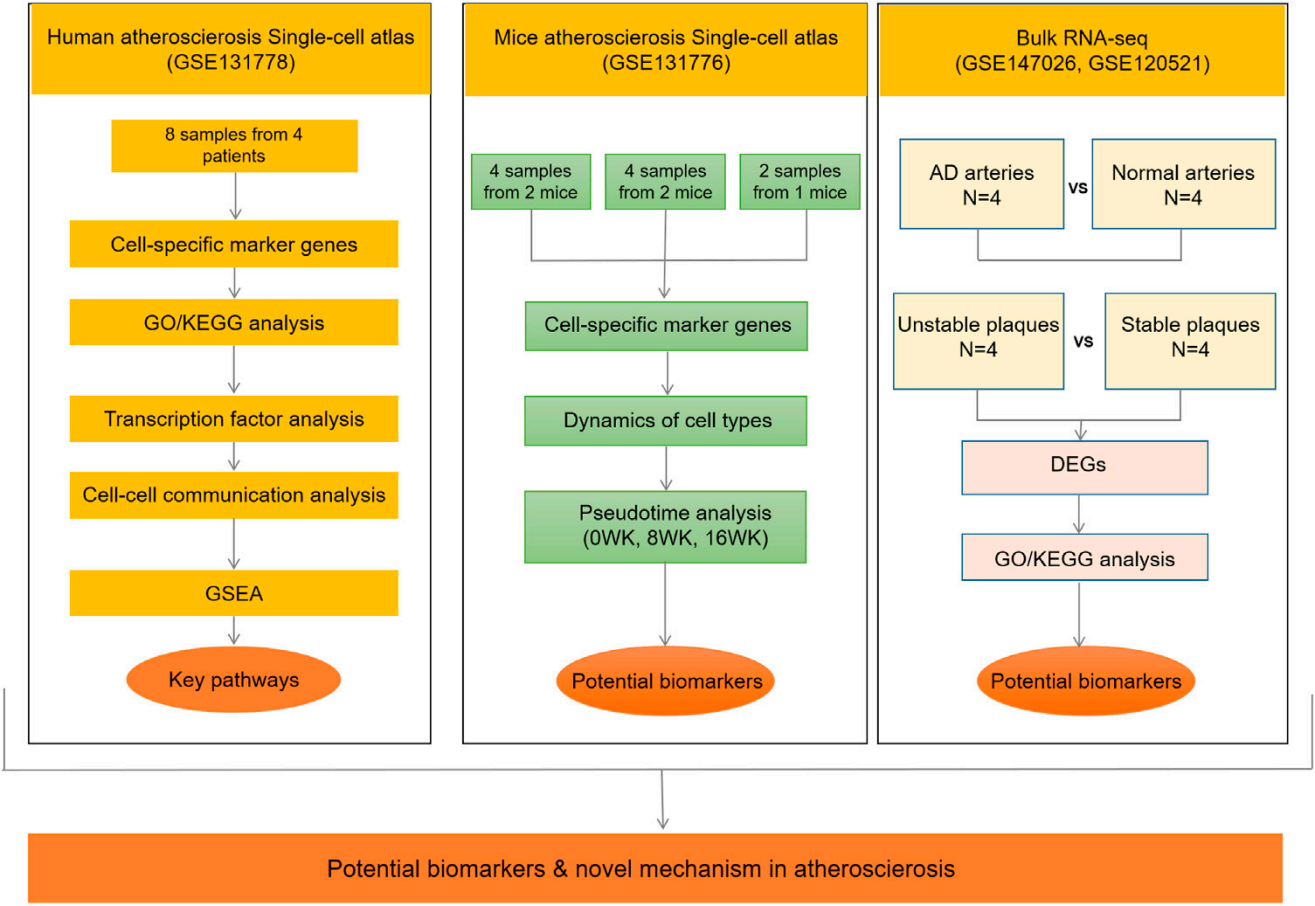
Flow diagram of the entire study design. AD, aortic dissection; DEGs, differentially expressed genes; GSEA, gene set enrichment analysis; 0WK: 0-week; 8WK: 8-weeks; and 16WK: 16-weeks. GSE131778 (right coronary artery), GSE131776 (aortic root and ascending aorta), GSE147026 (aortic media tissue), GSE120521 (carotid plaques).

In present study (Figure 1), to further clarify the dynamic mechanism of atherosclerosis, we studied the dynamic characteristics of different cell types and cell-specific expressed genes through analyzing single-cell sequencing data from atherosclerotic human coronary artery tissues and ApoE^−/−^ mice artery tissues. To understand the role of fibroblasts in the development of atherosclerosis and the progress of fibrous plaques formation and rupture, we accurately calculated the proportion of cell types in different atherosclerotic periods. We analyzed gene expressions of different cell types to harvest the potential biomarkers of atherosclerosis. Based on analyzing transcriptional regulatory factors and constructing cell-cell communication networks, we explored the interaction between fibroblasts and other cell types during the atherosclerosis. Moreover, to identify the mechanism of plaque stability, we further compared the differences of gene expressions between ruptured and stable arterial plaques by RNA-seq. In addition, the differences of gene expressions between normal and dissected arteries were also detected. Finally, we revealed several important pathways involved in regulating plaque formation and rupture using gene set enrichment analysis (GSEA).

## 2 Materials and methods

### 2.1 Study design

A flow diagram summarizing the entire study design is provided in Figure 1.

### 2.2 Single-cell RNA sequencing data analysis

#### 2.2.1 Acquisition of single-cell sequencing dataset

We obtained single cell sequencing dataset of human atherosclerotic coronary arteries (GSE131778) and mouse atherosclerotic aorta tissues (GSE131776) provided by previous researchers (Wirka et al., 2019) from the GEO database (https://www.ncbi.nlm.nih.gov/geo/). We also obtained single cell sequencing datasets through the GEO database, including human atherosclerotic carotid arteries and mouse atherosclerotic aortas (GSE155514), mouse atherosclerotic aortas (GSE216579), mouse aortas (GSE214413) provided by previous researchers (Pan et al., 2020; Cui et al., 2023; Dong et al., 2023). In the single-cell sequencing data of atherosclerosis ApoE^−/−^ mice, we performed independently analysis of 0-week, 8-weeks, and 16-weeks. The dataset was subsequently analyzed using R package Seurat (version 3.1.2) (Butler et al., 2018), including filtering, normalization, dimensionality reduction, clustering, and identification of cell types. In the single-cell sequencing data of mouse, we used the Kruskal-Wallis function in R to test the difference between normal, 8-weeks, 12-weeks, 16-weeks, and 22-weeks (Supplementary Figure S3D).

#### 2.2.2 Cell filtering, normalization and dimensionality reduction

**FIGURE 2:**
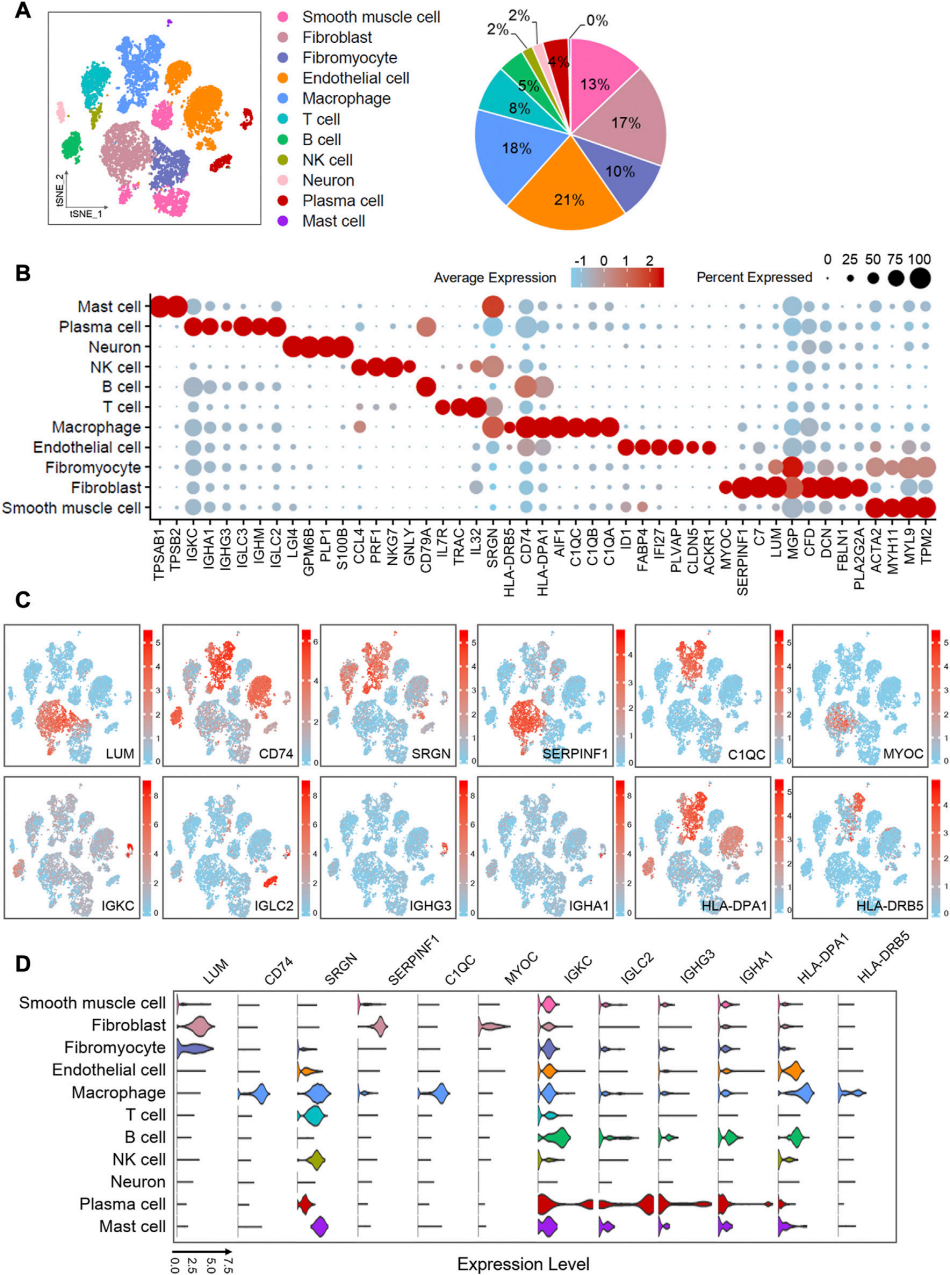
Single-cell atlas of human atherosclerotic coronary arteries. **(A)** t-SNE plots showing clusters of 11,754 cells from eight human atherosclerotic artery samples. Pie chart showing the proportions of cell types. **(B)** Dot plots showing z-score scaled average expression levels of top differentially expressed genes in each cell type. Differentially expressed genes are identified fromWilcoxon Rank Sum test (adj.p < 0.05). **(C)** t-SNE visualization of the expression of genes. **(D)** Violin plots showing loge (TPM+1) expression levels of genes. All of the expression levels are measuredwith the same scale. The analysis data comes from GSE131778 (GSM3819856, GSM3819857, GSM3819858, GSM3819859, GSM3819860, GSM3819861, GSM3819862, GSM3819863).

Cells expressing fewer than 500 genes were excluded, and genes expressed in fewer than 5 cells were removed. The remaining cells and genes were created as Seurat objects for further analysis. We discarded cells that express more than 3,500 genes as they might be low-quality “doublet.” At the same time, cells containing more than 7.5% of mitochondrial genes (MT-) were also discarded. After critical filtering, the data was normalized in the downstream analysis, multiplied by 10 000 and natural-log transformed into log_e_ (TPM+1) expression values. In the process of dimensionality reduction, we used the classic PCA and *t*-SNE algorithm. We obtained 1,500 highly variable genes from the log-transformed expression data, which were applied to execute the principle component analysis (PCA). We selected 20PCs from PC1 to PC20 for t-distributed stochastic neighbor embedding (*t*-SNE) analysis (Figures 2, 4; Supplementary Figures S2, S3).

#### 2.2.3 Clustering and identification of cell types

Cells clustering was performed with shared nearest neighbor (SNN) algorithm and *t*-SNE, and the resolution were set to 0.5 for human data, and 1.0 for mouse data (Figures 2, 4; Supplementary Figures S2, S3). We found the differential expressed genes (also called marker genes) of each cluster through the “FindAllMarkers” function, and these genes were used for cellular identification (Supplementary Tables S1, S3). We used known marker genes specifically expressed by cells to determine the cell type of each cluster. Then clusters were merged by cell type, and the differential expressed genes of each cell type were rediscovered for subsequent GO and KEGG pathway analysis (Supplementary Figure S1).

### 2.3 GO and KEGG pathway analysis

Based on differentially expressed genes (log_2_FC > 2, adj. *p* < 0.05 by Wilcoxon Rank Sum test) of all cell types identified above, we conducted biological progress and pathway enrichment analysis with Metascape (Zhou et al., 2019) (https://metascape.org/) (Supplementary Figure S1B). Furthermore, we performed GO and KEGG pathway analysis of marker genes for macrophages, fibroblasts, fibromyocytes, endothelial cells, and smooth muscle cells, using clusterProfiler package (v3.14.3) in R (Supplementary Figures S1C, D). We selected GO-enriched annotations that are closely related to atherosclerotic disease, and visualized significant results in R. The results of the significant enrichment of KEGG pathway were also visualized in R.

### 2.4 Transcription factor-target gene network analysis

**FIGURE 3:**
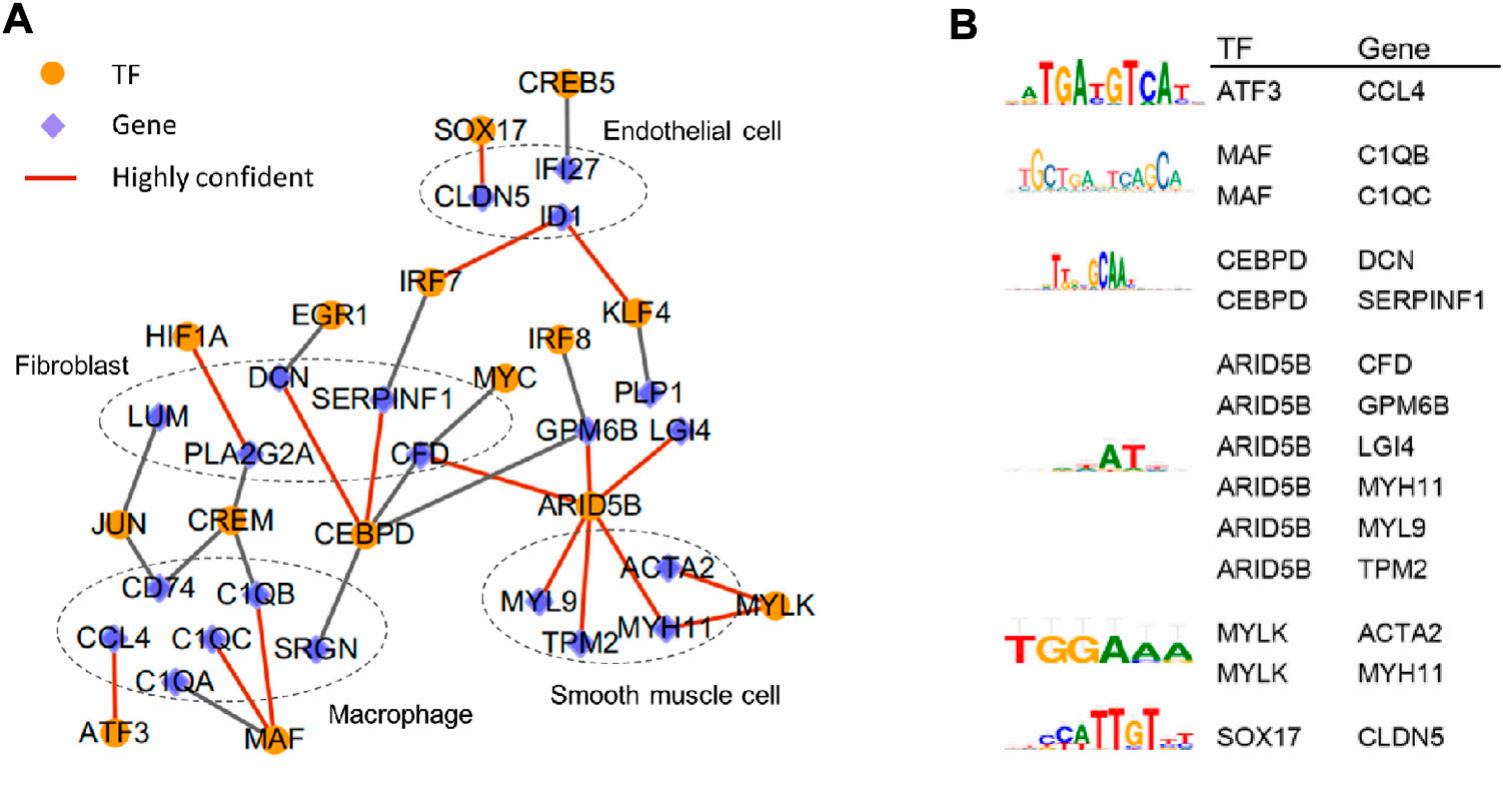
In-depth analysis of the correlation between various types of cells in human atherosclerotic coronary arteries. **(A)** Transcription factor-target gene network. Highly confident TFs and target genes are linkedwith red line. **(B)** The best binding motif of highly confident TFs and target genes. The data of this figure is from GSE131778.

We gathered 3,000 highly variable genes from macrophages, fibroblasts, fibromyocytes, endothelial cells and smooth muscle cells based on gene expression matrix of human single-cell sequencing dataset, including 2,066, 2,048, 1,178, 2,504, and 1,518 cells, respectively. These five types of cells were applied to predict transcriptional regulators independently, using the SCENIC (version 1.1.2-2) pipeline workflow (Aibar et al., 2017) in R (Figure 3A). After filtering genes and cells with the conditions of default parameters, we collected potential transcription factors (TFs) using GENIE3 (version 1.8.0) and selected potential regulons (direct binding targets) based on the RcisTarget database 1.6.0 (hg19 motif databases of 500 bp upstream and TSS centered 10 kb) (Figure 3A). Next, we used AUCell (version 1.8.0) to evaluate and rank the activities of all predicted transcriptional regulators and regulons (Figure 3B). Thus, highly reliable regulatory factors and corresponding optimal motifs were obtained. We used Cytoscape (version 3.2.1) to visualize the enriched transcription factor regulatory network between different cells (Figure 3A).

### 2.5 Cell-cell communication analysis

Cell-cell communication analysis was performed with 3,000 highly variable genes of all cells from human single-cell sequencing dataset using CellPhoneDB software (version 2.1.2) (Efremova et al., 2020) in python 3.7. We adopted statistical methods to analyze the expression of obtained ligand-receptor pairs among different cell types, and only pairs with significant difference (*p* < 0.05, one-sided Wilcoxon rank-sum test) in average expression levels were selected for subsequent analysis and visualization (Supplementary Table S2).

### 2.6 Pseudotime analysis

Before starting the pseudotime analysis, we extracted fibroblasts and fibromyocytes from the mouse single-cell sequencing datasets. After merging the data of three stages including 0 weeks, 8 weeks, and 16 weeks, we reconstructed a sub-dataset for single-cell trajectories analysis. In downstream analysis, Seurat (version 3.1.2) and Monocle2 package (version 2.14.0) (Qiu et al., 2017) in R were used for finding marker genes (log_2_FC > 1.5, adj. *p* < 0.05, Wilcoxon Rank Sum test) of cell clusters and ordering the cells by the expression of these genes. Finally, cells were divided into different states to imitate the differentiation of cells, and the expression dynamics of specific gene were visualized along the differentiation trajectories (Figure 5).

### 2.7 Bulk RNA-seq analysis

To learn more information, we performed bulk RNA-seq analysis of human atherosclerotic plaques (GSE120521) and human aortic dissection (AD) tissues (GSE147026). Plaques were obtained at carotid endarterectomy in symptomatic patients, and the visible area where the plaques rupture was dissociated and defined as unstable plaques. Genes with FDR (False Discovery Rate) values less than 0.05 were considered to have significant difference in dissection and normal arteries, and the results were visualized by volcano and heat maps (Figures 6A–C, Supplementary Figures S4A, B). In addition, we imported the FPKM (Fragments Per Kilobase per Million) expression values into R, which were obtained from RNA sequencing of human stable plaques and unstable plaques. Low-expression genes were dropped, and differential expression analysis was performed using the limma package (version 3.42.2) (Figure 6C). Similarly, we defined adj. *p* < 0.05 (Benjamini-Hochberg adjusted *p*-value) as the threshold for the significant differential expression of genes in stable and unstable plaques (Figure 6C).

### 2.8 Gene set enrichment analysis (GSEA)

We selected the first 3,000 highly variable genes from human atherosclerotic artery single cell sequencing data, and chose fibroblasts and fibromyocytes for gene set enrichment analysis using GSEA software (Subramanian et al., 2007). Cells of each type were sorted according to the specific gene expression level from high to low, and then divided into specific gene high expression group and low expression group. Then we compared the different expression patterns of these sorted data sets, with the guidance of the defined gene set from KEGG pathway analysis (Figures 6D, 6E; Supplementary Figure S4C).

## 3 Result

### 3.1 Single-cell atlas of human atherosclerotic coronary arteries revealed cellular components

The development of atherosclerosis and the process of plaque formation involve the participation of various cells. To understand the mechanism differences between all types of cells, we partitioned cellular clusters and constructed a single-cell map based on single-cell RNA sequencing data from human atherosclerotic artery tissues with severely pathological plaques. After critical quality filtering, we obtained 11 756 cells for the following graph-based clustering. We performed shared nearest neighbor (SNN) and t-distributed stochastic neighbor embedding (*t*-SNE), and divided these cells into 11 cell types (Figure 2A) by comparing differential expressed genes with the previous reported specific markers. Fibroblasts were identified on the basis of abundant lumican (*LUM*), decorin (*DCN*), type I collagen (*COL1A1* and *COL1A2*), and fibulin 1 (*FBLN1*). In particular, clusters that specifically express fibronectin 1 (*FN1*) were defined as “fibromyocytes,” which were reported to be fibroblast-like cells transformed from smooth muscle cells (Wirka et al., 2019). The clusters labeled “smooth muscle cell” were characterized with the high expressions of smooth muscle actin (*ACTA2*), myosin heavy chain 11 (*MYH11*), myosin light chain 9 (*MYL9*), and tropomyosin 2 (*TPM2*). We defined endothelial cells based on the expressions of platelet and endothelial cell adhesion molecule 1 (*PECAM1*), von Willebrand factor (*VWF*), fatty acid binding protein 4 (*FABP4*), claudin 5 (*CLDN5*), and interferon alpha inducible protein 27 (*IFI27*). With the guidance of immune-related factors, we distinguished five kinds of immune cells, including macrophages, T cells, B cells, NK cells, and mast cells. Macrophages specifically express C1q (*C1QA*, *C1QB* and *C1QC*), *CD74*, and *CXCL8*. The high expressions of C-C motif chemokine ligand 4 (*CCL4*) and C-C motif chemokine ligand 5 (*CCL5*) indicated the existence of NK cells. Due to the similar marker genes in T cells, B cells, and NK cells, we identified NK cells through the annotation of the original published report (Wirka et al., 2019) and the latest literature (Hudspeth et al., 2012). Tryptase alpha/beta 1 (*TPSAB1*) and tryptase beta 2 (*TPSB2*) were distinctively expressed in mast cells. T cells and B cells were distinguished by *CD3D* and *CD52*, respectively. Genes encoding immunoglobulin (*IGKC*, *IGHM*, *IGHA1*, *IGHG3*, *IGLC2*, and *IGLC3*) were detected in plasma cells. Neurons were classified in terms of the predominant expression of proteolipid protein 1 (*PLP1*), which encodes a transmembrane proteolipid protein (the predominant component of myelin). The expressions of the hallmarks used to annotate cell types are shown in a bubble chart (Supplementary Figure S1A).

To gain overall insights into the cellular composition of atherosclerotic arteries, we calculated the proportion of each cell type, which is shown in a pie chart (Figure 2A) along with *t*-SNE clustering plot. We found that endothelial cells accounted for the largest proportion, up to 21%. Followed by macrophages, fibroblasts, smooth muscle cells and fibromyocytes accounted for 18%, 17%, 13% and 10%, respectively. Endothelial cells are involved in the adhesion of leukocytes (Libby et al., 2011), which is the initial step in the formation of atherosclerosis. Moreover, when the atherosclerosis happens, it contributes to reduce vascular inflammation and inhibits disease development by autophagy (Zhang et al., 2020). Both regulation of immune metabolism by macrophages (Koelwyn et al., 2018) and accumulation of diseased macrophages (Robbins et al., 2013) play the key roles in the formation, growth, and eventual rupture of plaques. Fibroblasts and fibromyocytes accounted for 27% in total, indicating that they may play an important role in maintaining the physiological structure of arteries and promoting the pathological development of arterial disease. This is consistent with the previous reports (Sartore et al., 2001). Vascular smooth muscle cells are essential source of foam cells in atherosclerosis (Pi et al., 2021). The previous research has reported that smooth muscle cells could be transformed into unique “fibromyocytes” *in vivo* in mouse and human atherosclerotic lesions (Wirka et al., 2019), and the transformation is related to the pathogenesis of atherosclerosis. This resembles with a recent report that both the loss of smooth muscle cells inside the blood vessels and the significant increase in the amount of non-smooth muscle cells are related to calcification and inflammation of the aortic wall (Chen et al., 2020).

### 3.2 GO and KEGG pathway analyses of differential expressed genes indicated several potential biomarkers in human atherosclerosis

We obtained the differentially expressed genes for each cell population by cell grouping with log2FC > 2 and adj. *p* < 0.05 (Wilcoxon Rank and test), and we selected several differentially expressed genes that are most interesting to display in the bubble (Figure 2B). Furthermore, the biological significance of the above differential expressed genes were explored through gene ontology (GO) and pathway analysis using Metascape (Zhou et al., 2019) (Supplementary Figure S1B). We found that several biological processes related to atherosclerosis including humoral immune response, leukocyte migration, and lymphocyte proliferation were enriched, suggesting the critical role of immune system. In order to understand the biological function of various cells in atherosclerosis, GO gene ontology and KEGG pathway analyses were performed on cell-type-specific genes in smooth muscle cells, endothelial cells, fibroblasts, fibromyocytes, and macrophages (Supplementary Figures S1C, D). The data showed that *C1QA*, *C1QB*, and *C1QC* were involved in the complement coagulation cascade (hsa04610), *HLA-DPA1* and *CD74* were related to the antigen processing and presentation pathway (hsa04612), and *SERPINF1* was connected to the biological process of inflammatory response and cell motility.

We selected 12 more significant or newly discovered genes of interest from the differential expressed genes as the following research objects. *LUM* was mainly expressed in fibroblasts and fibromyocytes. *SERPINF1* and *MYOC* were enriched in fibroblasts. *CD74*, *SRGN*, *C1QC*, *HLA-DPA1*, and *HLA-DRB5* were abundantly expressed in macrophages. *IGKC*, *IGLC2*, *IGHG3*, and *IGHA1* were specifically expressed in plasma cells. At the same time, we also found that SERPINF1 is enriched in fibroblasts in another human atherosclerotic single-cell RNA-seq data analysis (Supplementary Figure S2; Supplementary Table S1). The *t*-SNE plots (Figure 2C) exhibited the distribution of these genes, and the violin plots (Figure 2D) showed their expression levels in various cells. Previous reports indicated that *LUM* was expressed in smooth muscle cells in human coronary atherosclerosis and promoted the formation of collagen fibers (Onda et al., 2002). *CD74* had also been reported as a new therapeutic target to reduce atherosclerotic inflammation (Martín-Ventura et al., 2009). C1q was involved in regulating macrophage molecular signals and inflammatory responses (Ho et al., 2016). Although the role of *SERPINF1* gene in atherosclerosis had not been reported yet, the pigment epithelium-derived factor (PEDF) encoded by *SERPINF1* gene had been widely reported in preventing atherosclerosis (Wen et al., 2017b; Wang et al., 2019b). Due to anti-inflammatory, anti-oxidation, anti-angiogenesis, anti-thrombosis, and anti-tumorigenic properties of PEDF, a large number of studies have been conducted in recent years, but the specific mechanism of the anti-atherosclerosis is still unclear. The current literature mainly focused on the expression of *SERPINF1* in smooth muscle cells and endothelial cells, but our data showed that *SERPINF1* was specifically expressed in fibroblasts. Our another data analysis also found that SERPINF1 was certainly expressed within human fibroblasts (Supplementary Figure S2). With more and more literature suggesting that fibroblasts play the important role in atherosclerosis, our finding will reveal a potential mechanism of *SERPINF1* in the atherosclerosis.

### 3.3 Transcription factor-target gene network revealed key transcriptional regulators in atherosclerosis

To investigate the transcriptional regulatory network underlying the main cell types, the transcription factor (TF) analysis was conducted based on single-cell expression matrix from human atherosclerotic coronary arteries using SCENIC(Aibar et al., 2017). 43 TFs including 19 in macrophages, 18 in endothelial cells, and 6 in fibroblasts were predicted. Subsequently, we focused on genes that were differential expressed between different cell types to construct transcription factor-target gene network (Figure 3A) and identify the best binding motif of highly confident TFs (Figure 3B). The regulatory network showed that some genes dominantly detected in the same cell type were regulated by the same transcription factor. Such as *DCN*, *SERPINF1*, and *CFD* highly expressed in fibroblasts were subject to *CEBPD*. *MAF* was in charge of *C1QA*, *C1QB*, and *C1QC*, which were mainly distributed in macrophages. It had been verified in ApoE (−/−) mice that the over expression of *DCN* could reduce inflammation in atherosclerotic plaques, thereby decelerating the progression of atherosclerosis (Al Haj Zen et al., 2006). Moreover, both genes specifically expressed in fibroblasts and genes predominantly expressed in macrophages are supervised by the same upstream TFs. For example, both *LUM* and *CD74* are driven by *JUN*. In addition, *IRF7* is responsible for *ID1* distributed in endothelial cells and *SERPINF1* in fibroblasts. Studies had also shown that *ID1* might affect the development of atherosclerosis through downregulation of low-density lipoprotein receptor (LDLR), uptaking lipids in endothelial cells (Zhang et al., 2018).

### 3.4 Cell-cell communication networks confirmed the key role of fibroblasts in maintaining tight connection between cell types in human atherosclerosis

To study the intercellular communication in arterial lesion tissues, we constructed cell-cell communication networks based on the single-cell RNA sequencing dataset of human atherosclerotic coronary arteries. We totally obtained 219 potential ligand-receptor pairs across 11 cell types using CellPhoneDB (Efremova et al., 2020) software, and then the significant ligand-receptor pairs (*p* < 0.05, one-sided Wilcoxon rank-sum test) were visualized the interaction networks according to the cell types (Supplementary Table S2). As the network shows, there are more ligand-receptor pairs among fibroblasts, fibromyocytes, macrophages, endothelial cells, and smooth muscle cells, compared with other cell types. We also found that the interactions of macrophages with other cell types were significant, and 30 ligand-receptor pairs were detected between fibroblasts and macrophages, indicating their key role in intercellular communication. Combined with the TF analysis, we speculated that fibroblasts played the vital role in atherosclerosis, and it may affect the biological changes of endothelial cells, macrophages, and smooth muscle cells.

### 3.5 Single-cell RNA sequencing analysis of mouse atherosclerotic arteries identified the dynamic changes of cellular components

**FIGURE 4:**
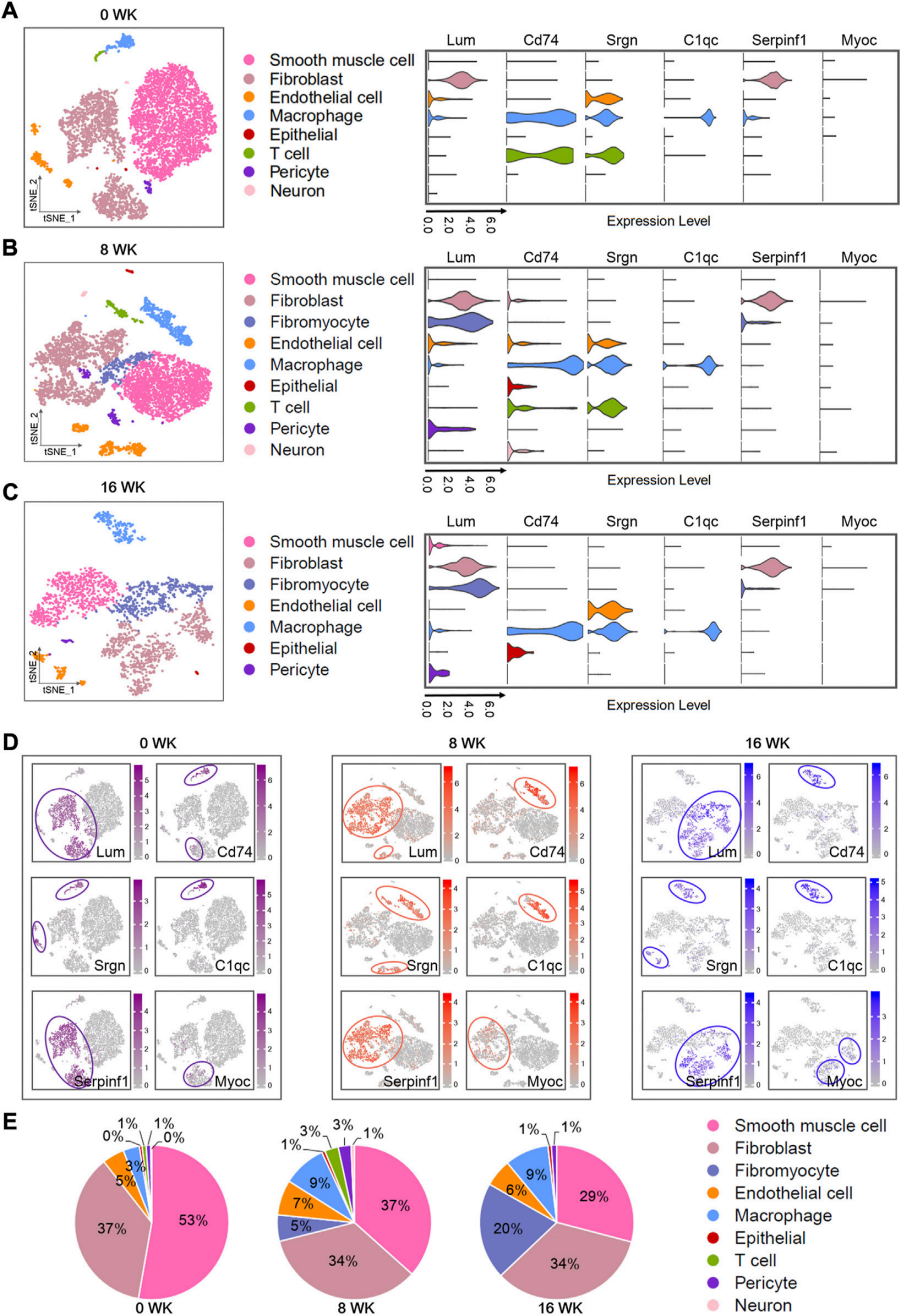
Single-cell atlas of mouse atherosclerosis arteries. **(A–C)** t-SNE plots showing different cell types in three stages (0WK: 0-week, 8WK: 8-weeks, and 16WK: 16-weeks) by scRNA-seq. Violin plots showing loge (TPM+1) expression levels of genes. All of the expression levels are measured with the same scale. **(A)** t-SNE plots of 6,836 cells from four 0-week mouse artery samples. **(B)** t-SNE visualization of 7,326 cells from four 8-weeks mouse artery samples. **(C)** t-SNE plots of 2,740 cells from two 16-weeks mouse artery samples. **(D)** t-SNE visualization of the expression of genes. **(E)** Pie chart showing the proportions of cell types. The analysis data comes from GSE131776, including 0WK (non-SMC: GSM3819844, GSM3819845; SMC: GSM3819850, GSM3819851), 8WK (non-SMC: GSM3819842, GSM3819843; SMC: GSM3819848, GSM3819849), and 16WK (non-SMC: GSM3819841; SMC:GSM3819846).

Considering that a simple advanced disease model did not reflect the dynamic changes of the proportion of various cells types and the expressions of differential genes during disease development, we conducted similar analysis of single-cell sequencing data from 0, 8, and 16 weeks ApoE^−/−^ atherosclerotic mice. According to published research, 0-week mice were defined as the baseline time (Wirka et al., 2019). ApoE^−/−^ mice having a high-cholesterol diet formed foam cells at 8-week, and formed advanced fibrous plaques at 15-weeks (Nakashima et al., 1994). Therefore, the three periods of mice can be a further explanation for the single-cell sequencing analysis of more severely human atherosclerotic lesions tissues. There were 6,836 cells retained after removing the low quality cells and genes in 0 weeks 7,326 and 2,740 cells were gathered in 8 weeks and 16 weeks, respectively. Globally, 9 main cell types were identified in three stages (Figures 4A–C) through supervising the expression levels of canonical cell-type-specific markers. Fibroblasts were identified by the high expression of *Pil6*, *Comp*, *Smoc2*, *Clec3b*, and *Chad*. Extracellular matrix (ECM) genes, such as *Col1a2*, *Lum*, and *Fn1* were abundantly expressed in fibromyocytes. Genes encoding actin, such as *Myh11*, *Myl9*, *Tpm2*, and *Acta2* were specifically expressed in smooth muscle cells. Endothelial cells were established on the basis of striking expressions of *Pecam1*, *Vwf*, *Fabp4*, and *Cldn5*. As expected, *C1qa*, *C1qb*, *C1qc*, and *Cd74* were predominantly expressed in macrophages. *Cd3g* and *Cd3d* were specifically expressed in T cells. Neuron significantly expressed *Plp1* and *Egf18*. In addition, pericytes were defined by the presence of *Notch3*. Epithelial cells were confirmed by the specific expression of *Upk3b* and *Cadm4*. Both pericytes (Juchem et al., 2010) and epithelial cells (Wilhelmson et al., 2018) had been reported in the pathological process of atherosclerosis.

We showed the expressions of several important genes in the violin diagram (Figures 4A–C) to explain their dynamic changes as the arterial disease develops. The specific distribution of these genes in various disease periods was also described in the scatter plots (Figure 4D). Since we had not defined plasma cells in the single-cell data of mice, the dynamics of related genes remained uncertain. We found that *Serpinf1* was expressed in fibroblasts and fibromyocytes at 8 and 16 weeks, and the expression was still dominant in fibroblasts. *Lum* was highly expressed in fibroblasts and fibromyocytes. *Myoc* was also mainly expressed in fibroblasts. *C1qc*, *Cd74*, and *Srgn* were highly expressed in macrophages. Moreover, a large amount of *Cd74* and *Srgn* were expressed in T cells, and abundant *Srgn* was also detected in endothelial cells. Meanwhile, Serpinf1 were also found to be expressed in fibroblasts of additional mice atherosclerotic single-cell RNA-seq data analysis (Supplementary Figures S3A–C; Supplementary Table S3), and was highly expressed at 8 and 22-weeks of atherosclerosis (Supplementary Figure S3D).

Thus, we severally analyzed the cell composition active of each cell type in three stages (Figure 4E). By calculating the ratio of each cell type in different periods (0-week, 8-weeks, and 16-weeks), we found smooth muscle cells (53%, 37%, and 29%), fibroblasts (37%, 34%, and 34%), endothelial cells (5%, 7%, and 6%), and macrophages (3%, 9%, and 9%) were still the dominant cell types, which was completely consistent with the above human results in our study. With the disease progressing, the proportion of fibromyocytes elevated significantly, but the ratio of smooth muscle cells declined obviously. We supposed that this disorder was strongly related to the pathological process of the disease, which might be caused by the phenotype modulation of smooth muscle cells into fibroblast-like cells. Our view was also supported by the report of Robert CW et al. (Wirka et al., 2019).

### 3.6 Pseudotime analysis of mouse single-cell sequencing data revealed the expression dynamics of potential biomarker in the development of atherosclerosis

**FIGURE 5:**
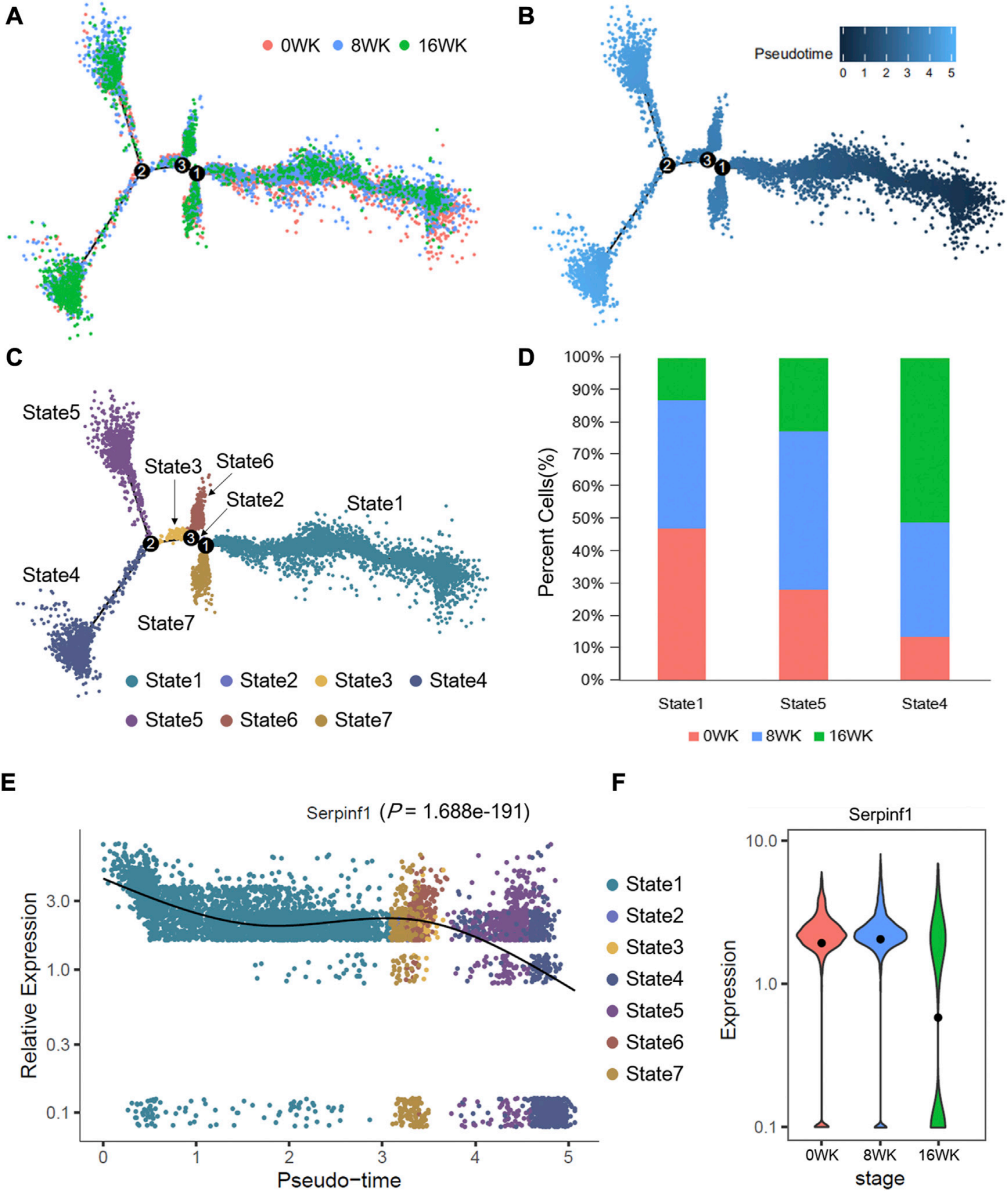
Pseudotime trajectories analysis of fibroblasts and fibromyocytes in mouse atherosclerosis arteries. Pseudotime trajectories of cells (6,811 cells in total) colored by **(A)** disease stages, **(B)** pesudotime value, and **(C)** cell states. **(D)** The proportion of cells in state1, state4, and state5. **(E)** The expression dynamics of *Serpinf1* is showing with the pseudo-time and cell states. *p*-value is by likelihood ratio tests. **(F)** The log_e_ (TPM+1) expression level of *Serpinf1* in three stages. The black dots represent the average expression level. The data of this figure is from GSE131776.

We sought to gain in-depth insights of the dynamic shifts of the above discovered potential biomarker through conducting further pseudotime analysis of single-cell sequencing dada. According to our study, *Serpinf1* was highly expressed in fibroblasts and fibromyocytes, and previous research also depicted that these cells had the important role in atherosclerosis. To obtain independent evidence of the expression dynamics of *Serpinf1* in fibroblasts and fibromyocytes, we constructed the predict single-cell differentiation trajectory according to the expression of marker genes, which was composed of pseudo-temporally ordered fibroblasts and fibromyocytes across the three stages (0-week, 8-weeks, and 16-weeks) (Figure 5A). Finally, 3 differentiation branch points were identified on the trajectory tree, and the cells were divided into 7 states (Figure 5C). Combined with the pseudotime of trajectory (Figure 5B), we concluded that state 1 was the initial state, and state 4 was the final state in the differentiation trajectory tree.

Notably, the expression of *Serpinf1* decreased in the pseudo-time trajectory, which was most noticeable in state 4 and state 5 (Figure 5E). We further analyzed the cellular components of state 1, state 4, and state 5. We found that cells at 0-week and 8-weeks mainly concentrated in state 1 and state 5. State 4 was enriched with cells at 16-weeks at the end of differentiation trajectory (Figure 5D). In addition, we analyzed the expression of *Serpinf1* in the three stages (Figure 5F). The results revealed the fact that *Serpinf1* experienced a significantly diminishing expression (*p* = 1.688e-191, likelihood ratio tests) with the progression of atherosclerosis. We speculated that the downregulation of *Serpinf1* might be a possible reason for the intensification of atherosclerosis. Previous studies had reported that PEDF encoded by *SERPINF1* gene was a biomarker of atherosclerosis (Tahara et al., 2011). It had been confirmed in both humans and mice model that PEDF played a protective role against atherosclerosis. PEDF could improve the stability of atherosclerotic plaques through inhibiting macrophage inflammatory response (Wen et al., 2017a), and the deficiency of PEDF accelerated the development of atherosclerosis by promoting the absorption of endothelial fatty acid (Wang et al., 2019a). These reports partly corroborate our suggestion, thus we also conclude that the downregulation of *Serpinf1* gene expression in fibroblasts and fibromyocytes results in a shortage of PEDF, which aggravate the development of atherosclerosis and accelerate the formation of plaques.

### 3.7 Bulk RNA-seq analysis of human atherosclerotic plaques and aortic dissection (AD) tissues considered the potential biomarkers

**FIGURE 6:**
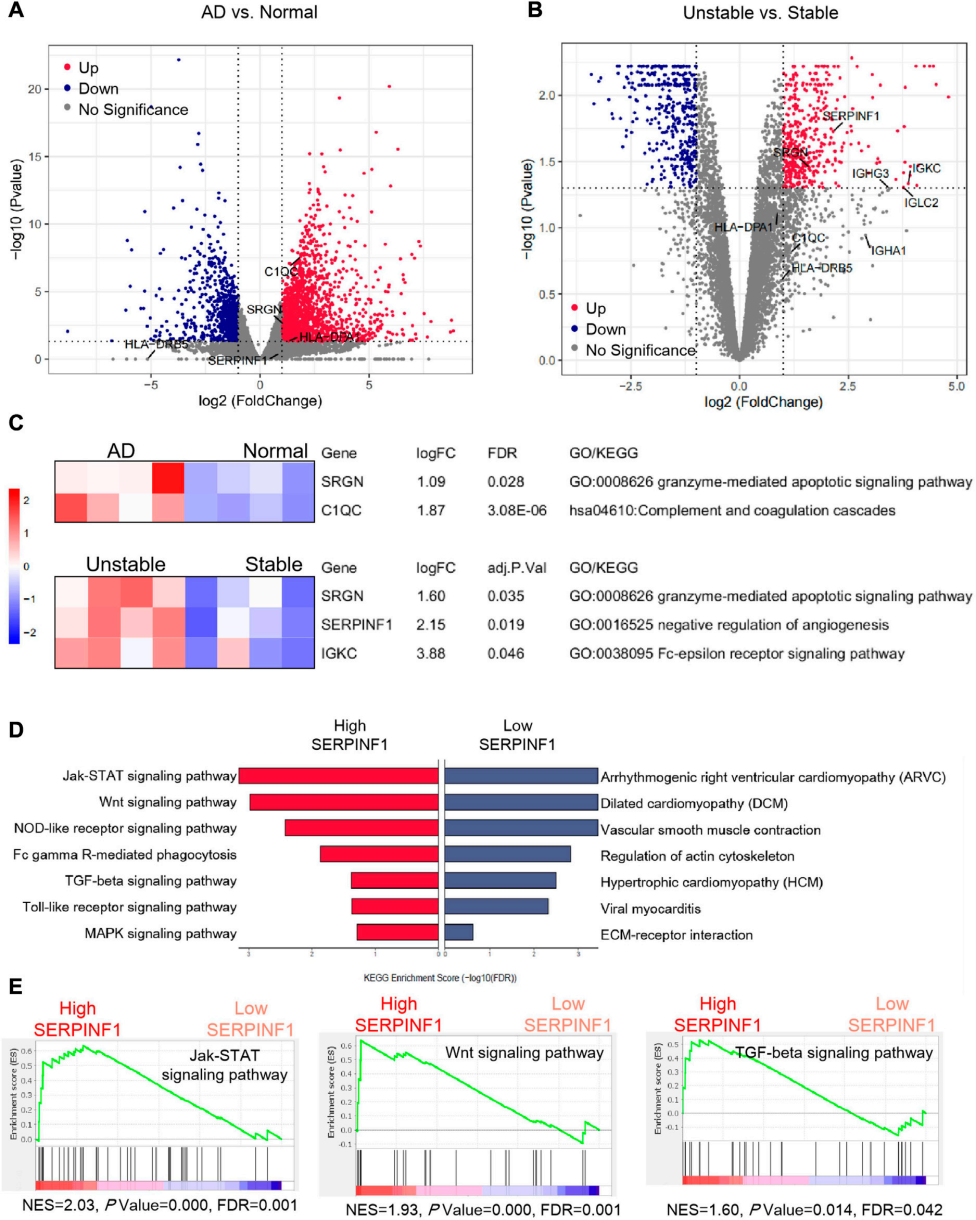
Bulk RNA-seq analysis and gene set enrichment analysis. **(A)** Volcano plot showing differential expression of genes in dissection tissues and normal tissues of arteries. Four normal human artery samples and four dissected human artery samples are used to identify DEGs (differentially expressed genes). **(B)** Volcano plot showing differential expression of genes in stable and unstable atherosclerotic plaques. Four stable human arterial plaque samples and four unstable human arterial plaque samples are compared to gain DEGs. The *p* values are by an empirical Bayes moderated t-statistics test, and adj. *P* represents Benjamini-Hochberg adjusted *p*-value **(C)** Heat map showing the z-score scaled relative expression levels of DEGs, and GO/KEGG annotations showing the related biological processes of DEGs. **(D)** The enrichment results of fibroblasts and fibromyocytes from eight human atherosclerotic artery samples using gene set enrichment analysis. **(E)** Important signaling pathways enriched by gene set enrichment analysis. *p* values are estimated by an empirical phenotype-based permutation test. The analysis data comes from GSE147026 (Normal arteries: GSM4412476, GSM4412477, GSM4412478, GSM4412479; AD arteries: GSM4412480, GSM4412481, GSM4412482, GSM4412483), and GSE120521 (Stable plaques: GSM3402504, GSM3402506, GSM3402508, GSM3402510; Unstable plaques: GSM3402505, GSM3402507, GSM3402509, GSM3402511).

Regarding gene expression differences between human atherosclerotic tissue and normal arterial tissue, the available sequencing data was scarce so far. Studies had shown that patients with severe atherosclerosis had a higher risk of Stanford type B aortic dissection (Barbetseas et al., 2008). Since atherosclerosis is a high risk factor for aortic dissection, we hope to further explore the possible relationship between atherosclerosis and arterial dissection. In order to verify whether the above potential biomarkers were specifically dysregulated in diseased tissues, we performed the analysis of RNA-seq data of human arterial dissection lesion tissues. Moreover, we also monitored the expression characteristics of potential biomarkers in stable plaques and unstable plaques (ruptured plaques), based on the RNA-seq data of human atherosclerotic carotid plaques. The volcano plot showed the differential expressions of genes (Figures 6A, B), and the heat map specifically describes the expressions of differential expressed genes in each sample (Supplementay Figures S4A, B).

We found that both *C1QC* and *SRGN* in the arterial dissection were obviously upregulated compared to the normal group, while the change of *SERPINF1* was not significant (Figure 6A). We speculated that the inflammatory response mediated by macrophages played an important role in aortic dissection. Our view was supported by previous study that increased macrophage recruitment and inflammatory response could promote blood vessel remodeling and pathological progress of dissection (Wang et al., 2018b). Compared with stable arterial plaques, *SERPINF1*, *SRGN*, and *IGKC* were evidently upregulated in unstable plaques (Figure 6B). Interestingly, this result was contrary to the downregulation of *Serpinf1* as the disease progresses that we previously obtained from the analysis of single-cell data in mice. We speculated that *SERPINF1* played the complicated role in preventing atherosclerosis. In the early stages of the disease, the downregulation of *SERPINF1* mediated the formation and development of the disease. In the advanced stage, the expression of *SERPINF1* was upregulated, which reduced the stability of plaques and promoted the rupture. However, there was no report about the role of *SRGN* and *IGKC* in unstable arterial plaques. We propose that the upregulation of *SRGN* may be a novel biomarker for unstable arterial plaques and arterial dissection, and the increase of *IGKC* in serum could also be considered as a new potential marker for the formation of severe unstable arterial plaques.

Further, we performed the related gene ontology (GO) annotations and KEGG pathway analyses of *SERPINF1*, *SRGN*, *C1QC*, and *IGKC* using DAVID (Huang et al., 2009) (https://david.ncifcrf.gov/) (Figure 6C). *SRGN* was involved in granzyme-mediated apoplotic signaling pathway (GO: 0008626). *C1QC* was related to complement coagulation cascades (hsa04610). *IGKC* was associated with Fc-epsilon receptor signaling pathway (GO: 0038095). *SERPINF1* participated in the negative regulation of angiogenesis (GO: 0016525).

### 3.8 Gene set enrichment analysis (GSEA) revealed the signaling pathways involved in atherosclerosis

Finally, we explored the possible signaling pathways involved in atherosclerosis. We performed gene set enrichment analysis of genes with specific expression pattern based on human single-cell data. The significant enrichment results (FDR<0.05) were shown with normalized enrichment score (NES) (Figure 6E; Supplementary Figure S4C). We detected that several signaling pathways were enriched in fibroblasts and fibromyocytes with high expression of *SERPINF1*, including Jak-STAT signaling pathway, Wnt signaling pathway, NOD-like receptor signaling pathway, TGF-beta signaling pathway, Toll-like receptor signaling pathway, and MAPK signaling pathway. Among them, the Jak-STAT signaling pathway is significantly enriched in fibroblasts expressing *SERPINF1*. Moreover, some common cardiovascular diseases were enriched in cells with low expression of *SERPINF1*, such as arrhythmogenic right ventricular cardiomyopathy (ARVC), dilated cardiomyopathy (DCM) and hypertrophic cardiomyopathy (HCM) (Figure 6D).

Our findings were supported by many literatures. In the model of atherosclerotic rabbits, the phosphorylation levels of JAK2 and STAT3 were increased, and the expression of SOCS3 was also upregulated, indicating that inhibition of JAK2/STAT3/SOCS3 signaling can alleviate atherosclerosis (Yang et al., 2020). The latest research indicated that lipopolysaccharide (LPS)-activated scavenger receptors in mouse macrophages were regulated by JAK-STAT-dependent pathways (Hashimoto et al., 2020). Studies had confirmed that Wnt/β-catenin signaling caused VSMC proliferation during intimal thickening in atherosclerosis (Tsaousi et al., 2011). Rui Wang et al. (Wang et al., 2018a) reported that the activation of NLRP3 (Nod-like receptor family) inflammatory bodies promoted the formation of foam cells in vascular smooth muscle cells. There are many reports about TGF-beta signaling pathway in atherosclerosis. TGF-beta1 is an anti-atherosclerotic cytokine with vascular protection. The over expression of TGF-beta1 impeded the progress of plaque growth, stabilized plaque structure, and prevented aortic dilation (Frutkin et al., 2009). In addition, *in vivo* experiments in mice have also verified the important role of Toll-like receptor signaling in atherosclerosis (Karadimou et al., 2020).

## 4 Discussion

In this study, the in-depth analysis of four datasets (GSE131778, GSE131776, GSE120521, and GSE147026) were conducted. We found that *SERPINF1*, which was highly expressed in fibroblasts, participated in the regulation of atherosclerosis through various signaling pathways. We proposed a potential mechanism, *SERPINF1* regulated the development of atherosclerosis and plaques stability through Jak-STAT signaling pathway.

Through the analysis of single-cell sequencing dataset of human atherosclerotic arteries, we found that fibroblasts, fibromyocytes, endothelial cells, macrophages, and smooth muscle cells accounted for more than 90% in total. Through analysis of biological processes and signaling pathways involved in cell-specific differential expressed genes, we found several potential atherosclerosis biomarkers, including *SERPINF1*, *SRGN*, *HLA-DPA1*, *HLA-DRB5*, *IGKC*, *IGLC2*, *IGHG3*, and *IGHA1*. Previous reports on *SERPINF1* had focused on endothelial cells (Ma et al., 2018; Wang et al., 2019b) and smooth muscle cells (Ma et al., 2018), but our results indicated that *SERPINF1* was more abundantly expressed in fibroblasts. Recent studies on single-cell RNA-seq of atherosclerosis also suggest the availability of fibroblast, suggesting that fibroblast may be another important cell population in the induction and progression of atherosclerosis (Pan et al., 2020; Cui et al., 2023; Dong et al., 2023). Moreover, transcription factor analysis indicated that there were common regulatory factors among fibroblasts, macrophages, endothelial cells, and smooth muscle cells. Cell-cell communication analysis revealed the important role of fibroblasts in intercellular communication. Further, we conducted an in-depth analysis on ApoE^−/−^ atherosclerotic mice to clarify the dynamics of *SERPINF1* in the development of the disease. We confirmed that *SERPINF1* was mainly expressed in fibroblasts and fibromyocytes. The proportion of fibromyocytes increased with the development of atherosclerosis, revealing its important role in the pathological process. The subsequent pseudotime trajectory analysis of single-cell data of mice found that the expression of *SERPINF1* in fibroblasts and fibromyocytes showed a significantly downward trend with the disease progressing. However, RNA-seq data analysis showed that the expression of *SERPINF1* was increased in unstable plaques compared with the stable plaques. In this regard, we try to explain it from two aspects. Firstly, the single-cell sequencing data was derived from early-stage atherosclerotic mice with foam cells appeared at 8 weeks and fibrous plaques emerged at 15 weeks (Nakashima et al., 1994), while human RNA-seq data was derived from advanced atherosclerosis plaques (Mahmoud et al., 2019). Secondly, we also believed it was probably due to the sequencing samples from different tissues, and the single-cell sequencing samples of mice were obtained from arterial tissues of aortic root and ascending aorta (Wirka et al., 2019), while the human RNA-seq samples were obtained from plaques (Mahmoud et al., 2019). Therefore, as the samples from different disease stages and tissues, we obtained different results. Furthermore, through gene set enrichment analysis (GSEA) based on human single-cell dataset, we found that *SERPINF1* was involved in multiple signaling pathways, including Jak-STAT signaling pathway, Wnt signaling pathway, NOD-like receptor signaling pathway, TGF-beta signaling pathway, Tol-like receptor signaling pathway, and MAPK signaling pathway. So far, no study had reported that *SERPINF1* was involved in the regulation of atherosclerosis through the Jak-STAT signaling pathway. However, in our enrichment results, Jak-STAT signaling pathway was enriched the most significantly.

During the intensification of atherosclerosis and the formation of plaques, the increase in the proportion of fibroblast-like cells was an important sign (Supplementary Figure S4D), which might be caused by the smooth muscle cell phenotypic modulation (Wirka et al., 2019) and endothelial mesenchymal transformation (Evrard et al., 2016). Another reasonable explanation was that the adventitial fibroblasts translocated to the intima after arterial injury, accompanied by a phenotypic transition to myofibroblasts (Shi et al., 1996). Moreover, we believed that *SERPINF1*, which is mainly expressed in fibroblasts and fibromyocytes, regulated atherosclerosis through participating in Jak-STAT signaling pathway, Wnt signaling pathway, and TGF-β signaling pathway (Supplementary Figure S4E), which were the crucial mechanisms for plaque formation and rupture mediated by multiple cell types. Our results are supported by many previous studies. It had been reported that TGF-β signaling strength promoted the transcription, expression and secretion of PEDF, thereby inhibiting angiogenesis in systemic sclerosis (Liakouli et al., 2018). PEDF inhibited Wnt pathway-mediated fibrosis in renal epithelial cells (He et al., 2017). PEDF reduced the expressions of TGF-β1 and FN by inhibiting the phosphorylation of JAK/STAT, thereby exerting an anti-fibrotic effect in diabetic nephropathy (Mao et al., 2013). PEDF downregulated the expression of vascular endothelial growth factor (VEGF) by inhibiting the activation of JAK2/STAT3, providing a therapeutic strategy for preventing the development of diabetic complications (Zheng et al., 2010). We proposed that a similar mechanism might exist during atherosclerosis, as shown in Supplementary Figure S4E. In addition, the participation of multiple cell types was also considered in the pathological process of atherosclerosis. We speculated that TGF-β promoted the transcription and translation of *SERPINF1* in fibroblast, and increased the expression and secretion of PEDF. PEDF hindered the activation of Jak-STAT signaling pathway and Wnt signaling pathway in various cells, thereby jointly inhibiting vascular remodeling.

As a downstream mediator of various cytokines, hormones, and growth factors, JAK/STAT signal was an important intracellular signal transduction pathway (Rawlings et al., 2004). JAK activation could stimulate cell proliferation, differentiation, cell migration, and apoptosis (Rawlings et al., 2004). Several reports had proved that JAK/STAT signal transduction contributed to the expression of inflammatory genes (Ortiz-Muñoz et al., 2009; Qin et al., 2014), the apoptosis of macrophages (Lim et al., 2008), and the phenotypic activation of endothelial cells (Qin et al., 2014) in atherosclerosis. We speculated that the activation of the JAK/STAT signal pathway in atherosclerosis promoted the migration and phenotypic modulation of endothelial cells and smooth muscle cells, increasing the expression of inflammatory genes in macrophages. Recent reports have demonstrated that the inhibition of JAK/STAT signaling alleviated atherosclerosis in both ApoE^−/−^ mouse (Tang et al., 2020) and rabbit atherosclerosis models (Yang et al., 2020). A study of human arterial tissue also showed that blocking JAK2/STAT3 pathway could attenuate the progress of abdominal aortic aneurysm (Xiao et al., 2020). Reports of diabetic complications had shown that the pigment epithelium-derived factor (PEDF) encoded by *SERPINF1* gene was involved in the inhibition of JAK/STAT activation (Zheng et al., 2010; Mao et al., 2013). Thus, we proposed that *SERPINF1*, which was abundantly expressed in fibroblasts, involved in the pathological process of atherosclerosis through preventing the activation of Jak-STAT signaling. On the one hand, the downregulation of *SERPINF1* reduced the inhibition of Jak-STAT signaling, causing vascular remodeling, thereby compensating for the early endothelial injury and accelerating the progression of atherosclerosis. On the other hand, excessive plaques formed and gathered at an advanced stage of atherosclerosis. The upregulation of *SERPINF1* in unstable plaques reduced the content of collagen fiber and promoted the rupture of arterial fibrous plaques by inhibiting Jak-STAT signaling pathway. Previous reports had similarly confirmed that reduced fibrosis, reduced collagen content, and thinning of the fiber cap caused unstable plaques (Lutgens et al., 2008; Higashi et al., 2016).

*SRGN* binds to *CD44* receptor to participate in the inflammatory response and induce human chondrocyte damage in arthritis (Scuruchi et al., 2019). We suggest that the upregulation of *SRGN* is a novel biomarker for unstable arterial plaques and arterial dissection. Our biological process analysis results indicated that *SRGN* participated in the granzyme-mediated apoplotic signaling pathway, suggesting that it might be related to the apoptosis of immune cells in atherosclerosis, such as macrophages, T cells, and NK cells. In our findings, *IGKC* was mainly expressed in plasma cells, and its expression was significantly upregulated in unstable plaques. Biological function analysis revealed that *IGKC* might participate in Fc-epsilon receptor signaling pathway. It was previously reported that the activation of antigen-specific IgE bound by Fc epsilon receptor (FCERI) leaded to the release of potent inflammatory mediators, which played an important role in inflammation and allergic reactions (Kinet, 1999). We proposed that *IGKC* was involved in the inflammatory response in atherosclerosis and could be regarded as a novel biomarker for the formation of severe unstable arterial plaques.

In this study, we had combined multiple datasets to comprehensively analyze the dynamic changes in cell composition and gene expression at various stages of the disease. Our research indicates that *SERPINF1* is related to the regulation of arterial plaque formation and rupture, and reveal that the expression of *SERPINF1* in fibroblasts and fibromyocytes changes dynamically as the disease worsens. In addition, we also elaborated several potential signaling pathways that *SERPINF1* involved in the regulation of plaque formation and rupture, including Jak-STAT signaling pathway, Wnt signaling pathway, and TGF-β signaling pathway. Furthermore, a variety of cells including fibroblasts, smooth muscle cells, endothelial cells, and macrophages participated in this process. However, further experiments are still needed to prove our findings.

## Data Availability

The original contributions presented in the study are included in the article/Supplementary Material, further inquiries can be directed to the corresponding author.

## Supplementary material

SUPPLEMENTARY FIGURE S1 GO and KEGG analysis of differentially expressed genes. **(A)** Dot plot showing the z-score scaled average expression levels of hallmarks used for identification of cell types. **(B)** The gene ontology (GO) and pathway analysis results of differentially expressed genes using Metascape. **(C)** Circle plot showing the GO annotations related to atherosclerosis. **(D)** The pathways and genes enriched by KEGG analysis. The p.adjust represents Benjamini-Hochberg adjusted *p* value.

SUPPLEMENTARY FIGURE S2 Single cell data set analysis of arterial tissue in atherosclerotic human. **(A)** t-SNE plots showing cell type clusters of arterial tissue from atherosclerotic human. The fibroblasts are circled in red. **(B)** Violin plots of markers differentially expressed in cell type clusters. **(C)** Feature plots showing the global expression level of marker genes (Dcn,Col1a1,Col3a1) and SERPINF1 in the fibroblasts. The fibroblasts are circled in red. The analysis data comes from GSE155514 (GSM4705589, GSM4705590, GSM4705591).

SUPPLEMENTARY FIGURE S3 Single cell data set analysis of arterial tissue in atherosclerotic mice. **(A)** t-SNE plots showing cell type clusters of mice on normal and western diet. The fibroblasts are circled in red. **(B)** Violin plots of markers differentially expressed in cell type clusters. **(C)** Feature plots showing the global expression level of marker genes (Dcn,Col1a1,Col3a1) and Serpinf1 in the fibroblasts. The fibroblasts are circled in red. **(D)** Histogram of Serpinf1 gene expression in atherosclerotic mice at different periods. *p* value is calculated by Kruskal-Wallis test. The analysis data comes from GSE216579 (12WK:GSM6680846), GSE214413 (Normal aorta: GSM6605449; 16WK:GSM6605451), GSE155514 (8WK: GSM4705600, GSM4705601; 16WK: GSM4705603, GSM4705604; 22WK: GSM4705605, GSM4705606).

SUPPLEMENTARY FIGURE S4 Bulk RNA-seq analysis and gene set enrichment analysis. **(A,B)** Heat map showing the z-score scaled relative expression levels of differentially expressed genes. **(C)** Signaling pathways enriched by gene set enrichment analysis. P values are estimated by an empirical phenotype-based permutation test. **(D)** A schematic diagram to explain the increase in the proportion of fibroblast-like cells. **(E)** Simple mechanism diagram reveals that SERPINF1 participates in arterial plaque formation and rupture by regulating vascular remodeling.
