# Supplementary material for: Integrated single-cell RNA-seq analysis reveals the vital cell types and dynamic development signature of atherosclerosis
Source: Front Physiol. 2023 Mar 30;14:1118239. doi: 10.3389/fphys.2023.1118239 (PMC10117136; doi:10.3389/fphys.2023.1118239)
Supplement: Supplementary file 4 [file Image1.pdf]

Supplemental figures and legends

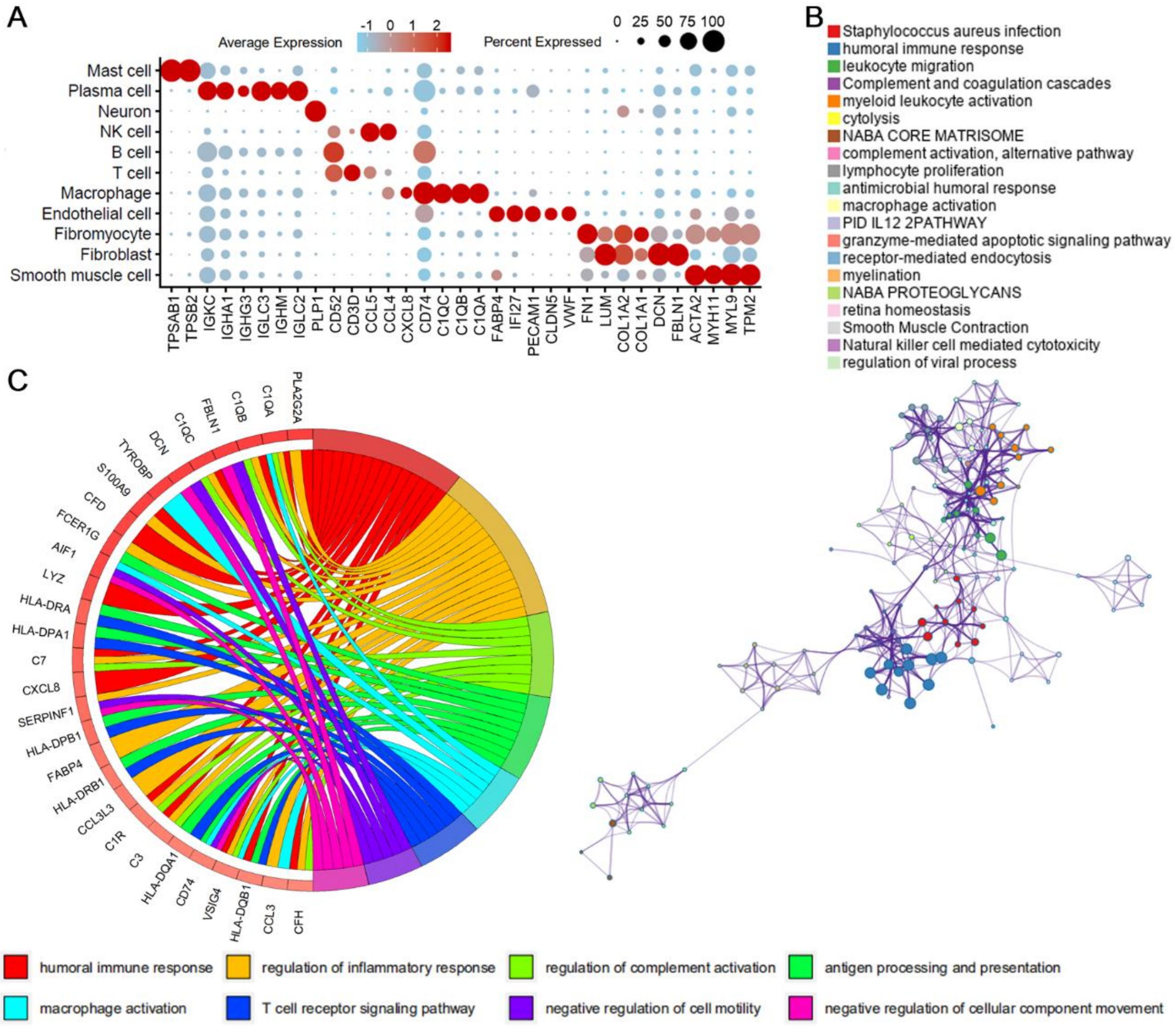

D

ID	Pathway	p.adjust	Enriched genes
hsa04610	Complement and coagulation cascades	7.08E-09	VSIG4/C1QC/C1QB/C1QA/CFH/CFD/C7/C3/C1R
hsa04612	Antigen processing and presentation	7.33E-08	HLA-DRB1/HLA-DRA/HLA-DQB1/HLA-DQA1/HLA-DPB1/HLA-DPA1/CTSB/CD74
hsa04620	Toll-like receptor signaling pathway	8.40E-03	CXCL8/CD14/CCL3L3/CCL3
hsa04270	Vascular smooth muscle contraction	0.018	MYL9/MYH11/ACTA2/PLA2G2A

Supplementary Figure S1. GO and KEGG analysis of differentially expressed genes. (A) Dot plot showing the z-score scaled average expression levels of hallmarks used for identification of cell types. **(B)** The gene ontology (GO) and pathway analysis results of differentially expressed genes using Metascape. **(C)** Circle plot showing the GO annotations related to atherosclerosis. **(D)** The pathways and genes enriched by KEGG analysis. The p.adjust represents Benjamini-Hochberg adjusted p value.

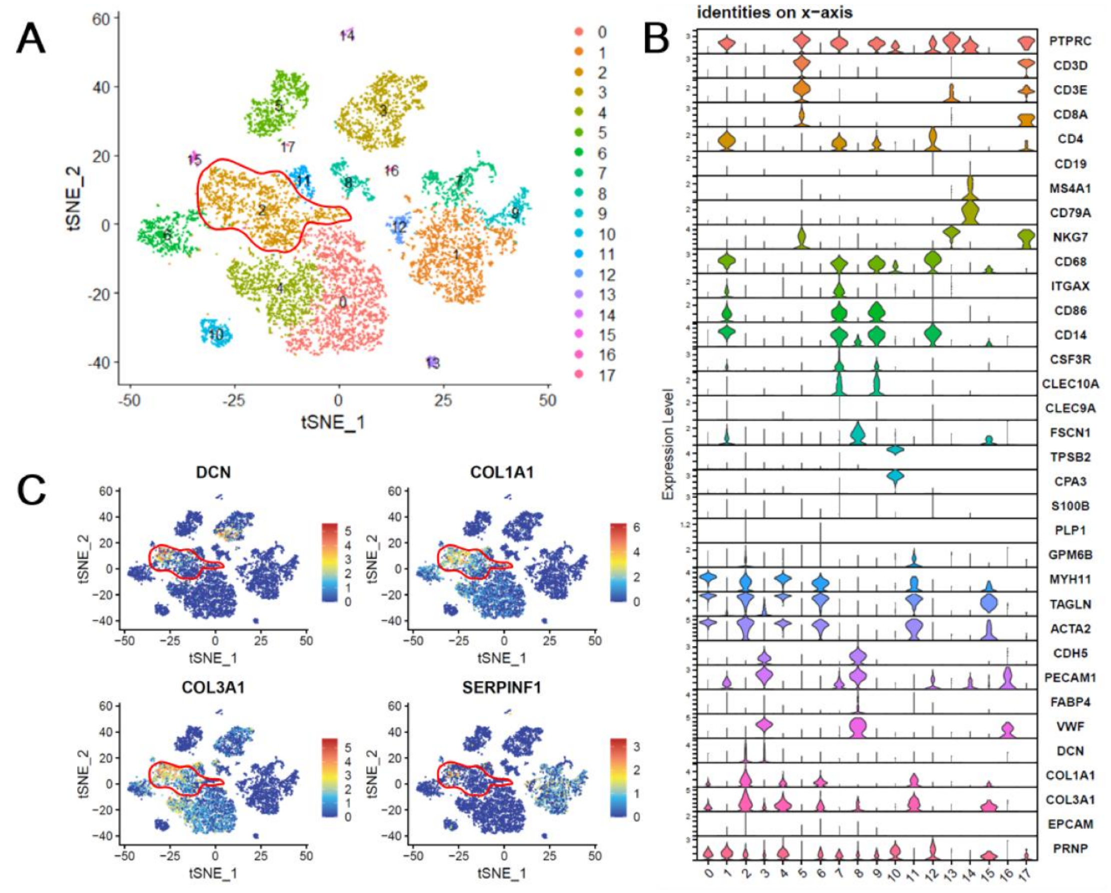

Supplementary Figure S2. Single cell data set analysis of arterial tissue in atherosclerotic human. (A) t-SNE plots showing cell type clusters of arterial tissue from atherosclerotic human. The fibroblasts are circled in red. **(B)** Violin plots of markers differentially expressed in cell type clusters. **(C)** Feature plots showing the global expression level of marker genes (Dcn,Col1a1,Col3a1) and SERPINF1 in the fibroblasts. The fibroblasts are circled in red. The analysis data comes from GSE155514 (GSM4705589, GSM4705590, GSM4705591).

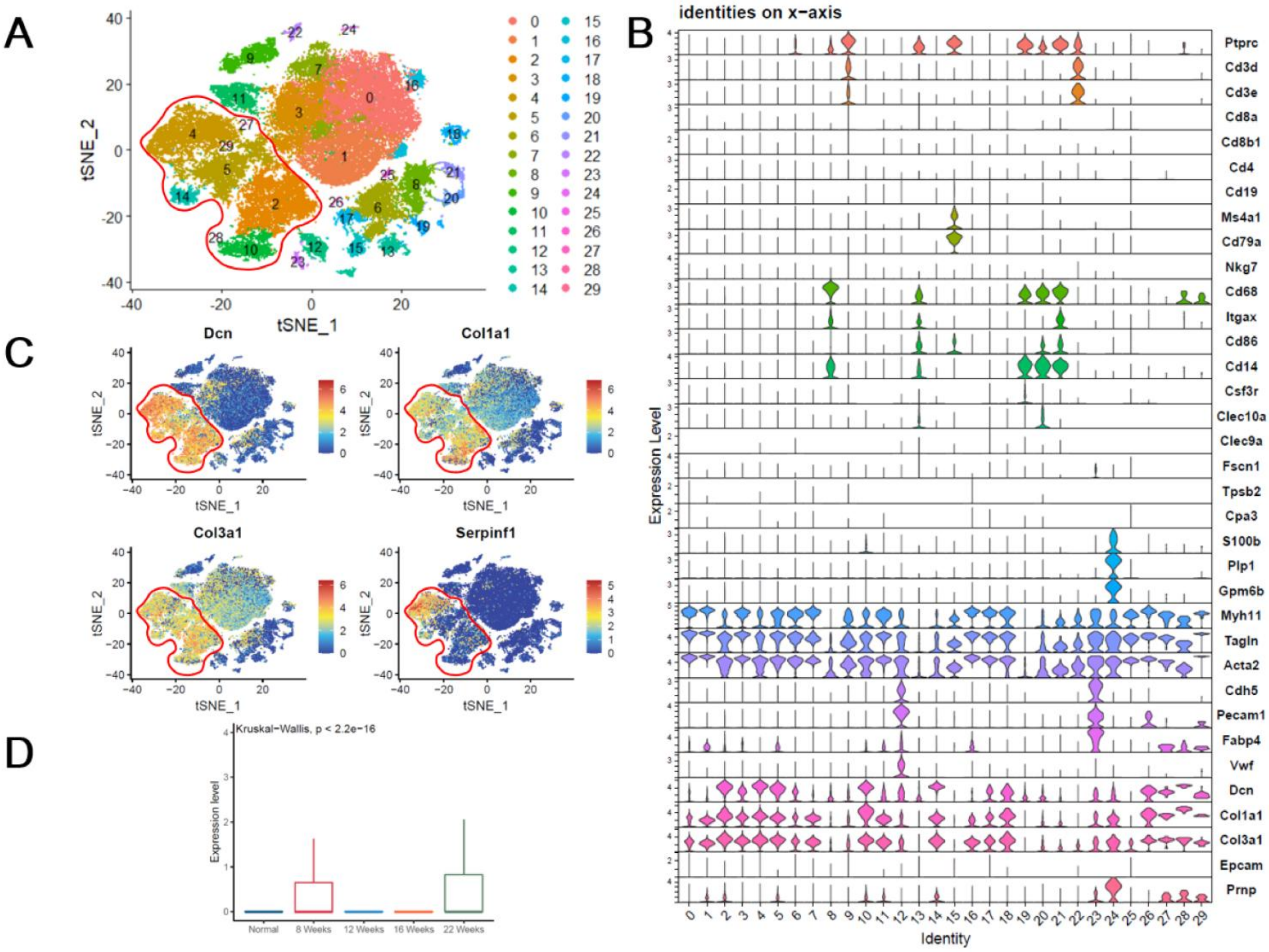

Supplementary Figure S3. Single cell data set analysis of arterial tissue in atherosclerotic mice. (A) t-SNE plots showing cell type clusters of mice on normal and western diet. The fibroblasts are circled in red. **(B)** Violin plots of markers differentially expressed in cell type clusters. **(C)** Feature plots showing the global expression level of marker genes (Dcn,Col1a1,Col3a1) and Serpinf1 in the fibroblasts.The fibroblasts are circled in red. **(D)** Histogram of Serpinf1 gene expression in atherosclerotic mice at different periods. P value is calculated by Kruskal-Wallis test. The analysis data comes from GSE216579 (12WK: GSM6680846), GSE214413 (Normal aorta: GSM6605449; 16WK:GSM6605451), GSE155514 (8WK: GSM4705600, GSM4705601; 16WK: GSM4705603, GSM4705604; 22WK: GSM4705605, GSM4705606).

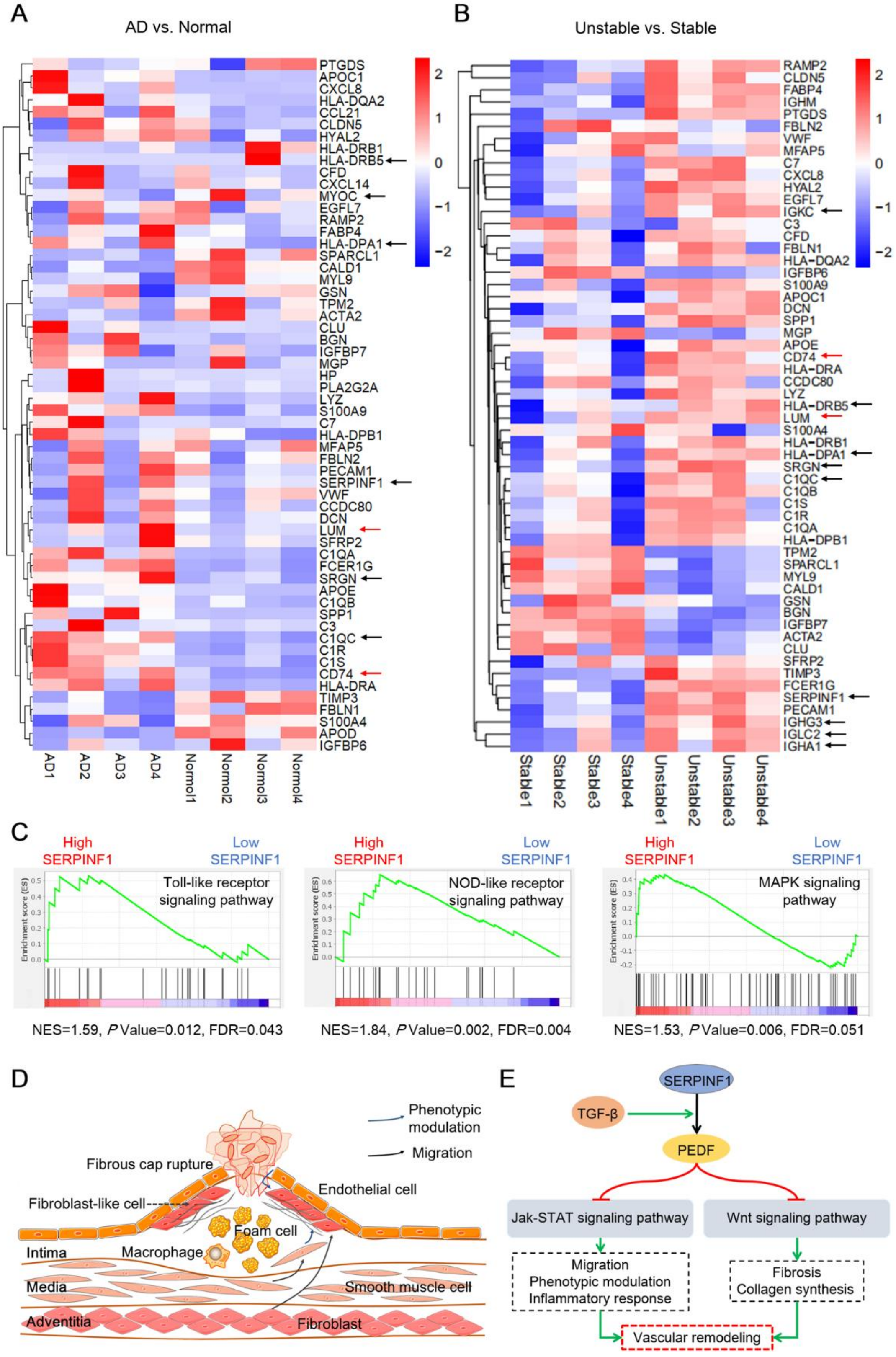

Supplementary Figure S4. Bulk RNA-seq analysis and gene set enrichment analysis. (A and B) Heat map showing the z-score scaled relative expression levels of differentially expressed genes. **(C)** Signaling pathways enriched by gene set

enrichment analysis. *P* values are estimated by an empirical phenotype-based permutation test. **(D)** A schematic diagram to explain the increase in the proportion of fibroblast-like cells. **(E)** Simple mechanism diagram reveals that *SERPINF1* participates in arterial plaque formation and rupture by regulating vascular remodeling.
